# Targeting FGF19/FGFR4 Pathway: A Novel Therapeutic Strategy for Hepatocellular Carcinoma

**DOI:** 10.3390/diseases3040294

**Published:** 2015-10-28

**Authors:** Dimitra Repana, Paul Ross

**Affiliations:** 1 Department of Medical Oncology, Guy’s and St. Thomas’ NHS Foundation Trust, SE1 9RT London, UK; E-Mail: dimitra.repana@gstt.nhs.uk; 2 Department of Oncology, King’s College Hospital, SE19 1RT London, UK

**Keywords:** hepatocellular carcinoma, FGF19, FGFR4, β-Klotho

## Abstract

Hepatocellular carcinoma (HCC) is a lethal cancer with limited systemic therapeutic options. Liver carcinogenesis is a complex procedure and various pathways have been found to be deregulated which are potential targets for novel treatments. Aberrant signalling through FGF19 and its receptor FGFR4 seems to be the oncogenic driver for a subset of HCCs and is associated with poor prognosis. Inhibition of the pathway in preclinical models has shown antitumour activity and has triggered further evaluation of this strategy to *in vivo* models. This review aims to describe the role of the FGF19/FGFR4 pathway in hepatocellular carcinoma and its role as a potential predictive biomarker for novel targeted agents against FGF19/FGFR4 signalling.

## 1. Introduction

In 2012 782,000 new cases of hepatocellular carcinoma (HCC) were reported worldwide from which 83% were in less developed countries and 50% just in China alone. HCC is the fifth most common cancer in men (554,000 new cases and 7.5% of all cancers) and the ninth most common cancer in women (228,000 new cases and 3.4% of all cancers). Prognosis is poor with an overall ratio of mortality to incidence of 0.95 resulting in 746,000 deaths in 2012 (9.1% of all cancer deaths) making it the second most lethal cancer after lung cancer [1].

Around 80%–90% of HCC is related to chronic inflammation and cirrhosis [2]. The major risk factors for HCC are Hepatitis B virus (HBV), Hepatitis C virus (HCV), alcoholic liver disease and non-alcoholic fatty liver (NAFLD) [3]. Despite proven efficacy of HBV vaccination in preventing HCC and improvements in antiviral treatment for both HBV and HCV it is estimated that worldwide at least 53% of HCC cases are attributed to HBV and 25% to HCV [4]. Increasing incidence of obesity and diabetes in Western countries is concerning due to its strong association with NAFLD which can lead to cirrhosis and HCC, although cirrhosis may not always precede [5]. It is estimated that 30% of the US population will be affected by NAFLD during their life introducing a potentially increasing high risk population [6].

Surgical resection and liver transplantation are indicated for patients with localized disease and are potentially curative treatments whilst transarterial chemoembolization (TACE) is used for patients where large and multifocal tumours who are not amenable to surgical treatments. However the majority of patients will relapse and will require systemic treatment where options are extremely limited since several chemotherapy and targeted agents have failed to show any meaningful outcomes [7,8]. Sorafenib is a multi-targeted tyrosine kinase inhibitor that interferes with tumour proliferation and angiogenesis by inhibiting the RAF kinase and the VEGFR mediated intracellular pathway. It is the only approved treatment based on the benefit seen in two phase III studies: the SHARP trial in a Western population showed a 3 month overall survival benefit *versus* best supportive care (10.7 *vs.* 7.9 months, 95% CI, 0.55–0.87, *p* < 0.001) [9] and a study in Asian-Pacific population showed an overall survival of 6.5 months for sorafenib *versus* 4.2 months for placebo (HR 0.68, 95% CI, 0.50–0.93, *p* = 0.014) [10].

Various signaling pathways involved in proliferation, cell differentiation, angiogenesis, invasion and metastasis are deregulated in HCC and are attractive targets [11,12,13]. Multikinase inhibitors with activity against angiogenesis and FGFRs have also been tried in HCC but failed to show superiority against sorafenib. Brivanib is a multikinase inhibitor with activity against VGFR1-3 and FGFR1-2. Despite promising results in II studies [14] it failed to meet its primary endpoints in phase III studies of patients with advanced HCC compared to sorafenib in the first line [15] and placebo in the second line setting [16]. Another multikinase inhibitor that has been tested was dovitinib with activity against VEGFR1-3, FGFR1-3 and PDGFR-β and similarly failed to show superiority against sorafenib in a phase II study of patients with advanced HCC [17]. Nintedanib which has activity VEGFR1-3, FGFR1-3 and PDGFRα and PDGFRβ has been approved for the treatment of non-small cell lung cancer and idiopathic pulmonary fibrosis [18]. When compared to sorafenib in patients with advanced HCC in a phase II study no significant differences were observed between the two agents in time to progression (TTP) and overall survival (OS) [19]. Notably all of these agents were used in unselected populations without any predictive biomarkers.

The need for better treatments in HCC is obvious and can only be achieved by better understanding of the molecular mechanisms that drive liver carcinogenesis. Evidence regarding the role of the members of FGF19-FGFR4 pathway in liver carcinogenesis is growing. Correlation with prognosis and *in vitro* experiments showing promising results with inhibition of the pathway have highlighted a subset of HCCs which are driven by FGF19/FGFR4 signaling and could potentially benefit from a selective inhibition of the pathway.

## 2. FGF Family

The Mammalian Fibroblast Growth Factor (FGF) family consists of 22 members which are further classified to 7 subfamilies according to phylogenetic analysis. They are named FGF1-FGF23 with FGFR15 not present in humans and FGF19 not present in mice and rats. FGF15 and FGF19 are considered orthologues and are usually referred as FGF15/FGF19. Each polypeptide growth factor consists of around 150–300 amino acids with a conserved core with 30%–50% identity. There are also four FGFR genes that encode the tyrosine kinase receptors and their variants resulting in seven major FGFR proteins (1b, 1c, 2b, 2c, 3b, 3c and 4). FGF signalling proteins are present in almost all tissues and are involved in various functions like embryogenesis, tissue repair, metabolism and neural function [20].

FGFs are also classified according to their cell-cell communication pattern as:

- Intracrine: they consist of the FGF11 subfamily (FGF11, 12, 13, 14). These factors are secreted intracellularly, they do not interact with FGFRs and their main role is regulation of the electrical excitability of neurons and other excitable cells like cardiomyocytes.
- Paracrine: they consist of the FGF1 subfamily (FGF1,2), the FGF4 subfamily (FGF4,5,6), the FGF7 subfamily (FGF3,7,10,22), the FGF9 subfamily (FGF9,17,18) and the FGF8 subfamily (FGF8,17,18). These are secreted proteins that bind to FGFRs and use heparin/heparan sulphate as a cofactor, although FGF1, 2, 3 can directly translocate to the nucleus and act as intracrine proteins. They are involved in embryogenesis and tissue repair by regulating cell proliferation, differentiation and survival.
- Endocrine: they consist of the FGF15/19 subfamily (FGF15/19, 21, 23). Endocrine FGFs have low affinity with heparin/heparin sulphate and they bind to FGFRs using the Klotho family proteins as cofactors. They act as hormones regulating bile acid, lipid and carbohydrate homeostasis [20,21,22].

FGF19 specifically is involved in bile acid synthesis, gallbladder filling, glycogen synthesis, gluconeogenesis, protein synthesis and reduction of adipose tissue [23]. Following a meal bile acids are secreted from the gallbladder to the small intestine and they activate Farnesoid X Receptor (FXR) which stimulates secretion of FGF19 from the ileum [24]. FGFR4 and β-Klotho, which is the main co-factor for FGF19 activation, are both highly co-expressed in the liver [25,26]. The FGF19-mediated signaling cascade results in downregulation of bile acid synthesis through inhibition of cholesterol 7a-hydroxylase (CYP7-A1) which is the rate-limiting enzyme for bile acid synthesis, suppression of cholecystokinin which promotes gallbladder emptying [21,24], promotion of glucogen and protein synthesis and inhibition of gluconeogenesis in liver [23]. FGF19 transgenic mice have reduced adipose tissue and low levels of cholesterol and triglycerides [27] and further data suggest that FGF19 is involved in inhibition of liposynthesis and increasing fatty acid oxidation by downregulating the activity of acetyl CoA carboxylase 2 [21]. Moreover FGF19 can promote proteinosynthesis by phosphorylating several proteins which promote translation [23].

**Figure 1:**
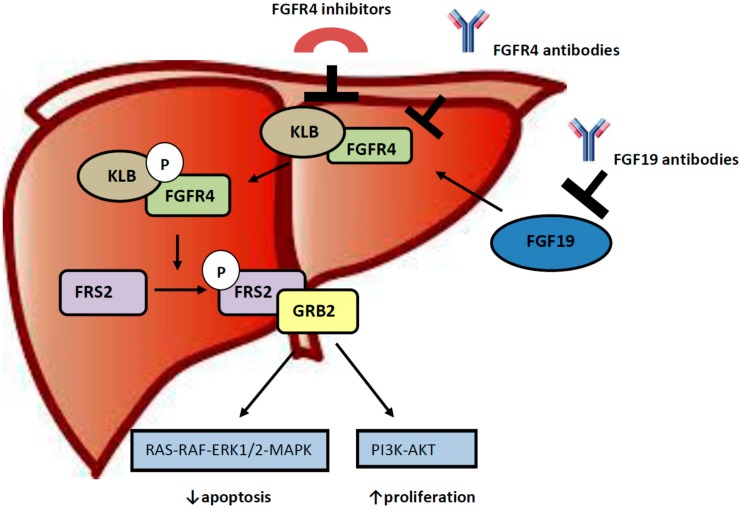
The FGF19/FGFR4 pathway with its main components and targets of inhibitory agents. FGF19: Fibroblast Growth Factor 19, FGFR4: Fibroblast Growth Factor Receptor 4, KLB: Klotho Beta, FRS2: Fibroblast growth factor Receptor Substrate 2, GRB2: Growth factor Receptor-Bound protein 2.

FGF19 has high specificity for FGFR4 and although it can bind to FGFR4 independently, the presence of β-Klotho which is a single-pass transmembrane protein and acts as a cofactor results in a more pronounced activation of FGFR4 and its phosphorylation [28]. Activation of FGFR4 results to phosphorylation of FGF receptor substrate 2 (FRS2) and recruitment of growth factor receptor-bound protein 2 (GRB2) and eventually activation of Ras-Raf-ERK1/2 MAPK and PI3K-Akt pathways which are involved in cell proliferation and anti-apoptosis [29] (Figure 1).

## 3. Role of FGF 19-FGFR4 Pathway in Hepatocellular Carcinogenesis

Evidence supporting the role of the FGF19/FGFR4 pathway in liver carcinogenesis is accumulating. Clearly FGF19 expression is significantly higher in hepatocellular carcinoma compared to non-malignant liver [30] and has oncogenic activity. FGF19-transgenic mice which overexpress FGF19 in skeletal muscle develop multiple hepatocellular carcinomas early in their life, usually by 10–12 months, whilst other tissues are not affected [31]. Miura *et al* showed that addition of FGF19 recombinant protein in hepatocellular cell lines increases proliferation and invasion and inhibits apoptosis whilst decreasing FGF19 and FGFR4 expression with small interfering RNA (siRNA) had the opposite results [30].

The rodent orthologue FGF15 also has mitotic and oncogenic activity. Following partial hepatectomy FGF15^−/−^ mice have increased intrahepatic bile acid levels and high mortality. Addition of cholestyramine and adenovirally delivered FGF15 resulted in improved survival. Improved outcomes following partial hepatectomy were also seen in FGF15^+/+^ mice suggesting that FGF15 induces hepatocellular proliferation and is important for liver regeneration [32]. Uriarte *et al* used diethylnitrosamine and CCl4 which are known to induce inflammation in liver and fibrosis-mediated carcinogenesis to both FGF15^−/−^ and FGF15^+/+^ mice. FGF15^+/+^ mice had higher tumour burden with more and bigger tumours and higher proliferation rate measured by Ki67 immunostaining supporting the oncogenic role of FGF15 [33].

FGF19 also seems to have a prognostic role for FGF1. Hyeon *et al* reported a series of 281 patients who underwent curative resection and found that 48% of the tumours expressed FGF19 protein and this was associated with larger size of the tumour (*p* < 0.0001), more advanced Barcelona Clinic Liver Cancer (BCLC) stage (*p* = 0.001) and early recurrence (*p* < 0.001) [34]. An association of FGF19 m RNA expression in the tumour with disease free survival and overall survival has also been reported [30].

The importance of FGFR4 in liver carcinogenesis has been showed both *in vitro* and *in vivo*. Gauglhofer *et al* showed that upregulation of FGFR4 *in vitro* promotes an aggressive phenotype of hepatocellular carcinoma by increasing invasion and tumourigenicity and downregulation of FGFR4 with si-RNA mediated knockdown or kinase-dead FGFR4 or soluble FGFR4 had the exact opposite results with decreased viability, invasion and tumour formation [35]. French *et al* bred FGF19 transgenic mice with FGFR4 knockout and found that only FGFR4 wild type mice could develop a hepatocellular carcinoma confirming that FGFR4 is necessary for FGF19-mediated carcinogenesis. They also used an FGFR4 neutralizing antibody which was created by immunizing FGFR4 knocked out mice with FGFR4 and showed that it had activity against hepatocellular carcinoma in mice and cell lines [36].

Different groups showed that approximately one third of HCCs have high FGFR4 expression. French *et al* used immunohistochemistry and found moderate to increased staining for FGFR4 for 33% of HCCs and confirmed this finding in a small number of 23 samples with qualitative real-time polymerase chain reaction (q RT-PCR) where FGFR4 expression was more than 2-fold higher in 30% of the tumours [36]. Similar results were reported from Ho *et al* who found that FGFR4 expression was significantly higher in 31.6% of HCCs (18/57 samples) [37]. The same group also reported an interesting association between a specific single nucleotide polymorphism (SNP) of FGFR4 (G388R) and alpha fetoprotein (AFP) which is an established clinical biomarker of tumour progression; using cell lines they proved that there is a direct link between them since stimulation of FGF19 results in elevation of AFP whilst silencing of FGFR4 results in reduced AFP production [37]. Presence of G388R polymorphism has also been associated with poor prognosis for head and neck cancers [38,39] and prostate cancer [40]. Furthermore expression of FGFR4 has been associated with higher TNM stage and shorter overall survival compared to no expression [41].

β- Klotho protein has been reported to be overexpressed (>2fold) in 25% of HCC samples and this finding was correlated with multifocal tumours. Unsurprisingly silencing β-Klotho resulted in decreased cell proliferation and suppression of the pathway. Interestingly, prolonged inhibition of the pathway resulted in an increased population of resistant cells with high expression of stem-cell markers like CD133 and CD44 [42].

Amplification of the 11q13 amplicon is one of the most common events in cancer and has a well-recognized role in carcinogenesis of several tumours including head and neck and breast cancers [43]. The 11q13 amplicon contains Cyclin D1 which is a known oncogene but also FGF19, FGF23 and ANO1 which are tumour promoting genes. 11q13 was found amplified in 14% of 89 HCC samples and 12 HCC cell lines as reported by Sawey *et al.* and RNAi-mediated knockdown of Cyclin D1 or FGF19 as well as use of a neutralizing FGF19 antibody resulted in tumour growth inhibition *in vitro* [44]. Genomic analysis with whole exome sequencing of 231 resected HCCs (HBV related 72%) identified a number of recurrent mutations, deletions and amplifications. FGF19 amplification was present in 5% of tumours and it was significantly associated with cirrhosis (*p* = 0.017) [45]. Based on all these results it has been suggested that amplification of 11q13 may represent a potential biomarker to select patients who will benefit from an antiFGF19 treatments [43].

## 4. Targeting the FGF19/FGFR4 Pathway

The recognition of the role of FGFRs in carcinogenesis lead to discovery of several FGFR inhibitors which are under investigation for different types of cancer including breast, bladder lung and liver cancer, cholangiocarcinoma and glioblastoma [46].

Pan-FGFR inhibitors are under evaluation in phase I-II trials like *NVP-BGJ398* [47], and *AZD4547* [48]. *FINN1*, *FINN2* and *FINN3* are covalent pan-FGFR inhibitors (FINN2 and FINN 3 are irreversible) [49]. However all of these agents have a much lower selectivity for FGFR4 compared to FGFR1-3. *JNJ-42756493* is another pan-FGFR inhibitor that showed acceptable toxicity profile in a phase I study; dose limiting toxicity was hepatotoxicity with grade 3 elevation of ALT/AST and the most common side effect was hyperphosphataemia which was seen in 60% of patients [50]. *LY2874455* is also a pan-FGFR inhibitor which had an acceptable toxicity profile in a phase I study with hyperphosphataemia being also common [51]. Hyperphosphataemia, nail changes and onycholysis, alopecia and hair modification, mucositis, dysgeusia and mucosal dryness, conjunctivitis, keratitis and eye dryness, asymptomatic retinal pigment epithelial detachment and osteoarticular pain, myalgias and muscle cramps seem to be FGFR-inhibition specific side effects whilst long term side effects are still unknown [46].

FGFR4 selective inhibitors are also under evaluation in an effort to increase specificity and reduce side effects. Several agents are under investigation. *FGF401* (Novartis) is a tyrosine kinase inhibitor with high selectivity against FGFR4 compared to FGFR1-3 and is under investigation in a phase I/II study in patients with hepatocellular carcinoma with FGFR4 and Klothoβ expression (ClinicalTrials.gov Identifier: NCT02325739). *AZ709* (Astra Zeneca) is a potent and selective inhibitor of FGFR4 with antitumour activity only in cell lines that expressed high levels of FGF19 and FGFR4 (m RNA and protein) [52]. *BLU9931* (Blueprint Medicines) is a potent and irreversible inhibitor of FGFR4. It is highly selective against FGFR4 because it forms a covalent bond with Cysteine 552 (Cys552) which is located near the ATP-binding site of FGFR4 and is unique for FGFR4 and not present in the other FGFRs. Similarly to previous reports *BLU9931* showed activity only in HCC cell lines with a fully functional FGF19/FGFR4/Klothoβ complex. Interestingly the three most sensitive cell lines (Hep 3B, HuH7 and JHH7) had a copy-number gain of FGF19. A robust tumour inhibition was also seen in mice treated with *BLU9931* with tumours that had an intact FGF19-FGFR4 pathway and treatment had an acceptable toxicity profile [53]. Blueprint Medicines has also developed another FGFR4 inhibitor called *BLU554* which is currently tested on a phase I trial (Clinical Trials Gov Identifier: NCT02508467). Finally *H3B6527* (H3 Biomedicine) is another highly selective FGFR4 inhibitor with potent antitumour activity in FGF19 amplified cell lines and mice. The agent was also tested in monkeys and interestingly was not associated with bile acid related side effects [54].

Another approach that has been studied is that of monoclonal antibodies against FGF19 or FGFR4. Desnoyers *et al* produced a neutralizing anti-FGF19 monoclonal antibody which prevented development of HCC in FGF19 transgenic mice that were treated with diethylnitrosamine which can accelerate tumour progression [55]. The safety of this antibody was tested in cynomolgus monkeys which have similarities in bile acid synthesis and cholesterol metabolism with humans and treatment with anti-FGF19 antibody was associated with increased bile acid synthesis and ileal malabsorption resulting in diarrhoea [56]. LD1 is a neutralizing antibody against FGFR4 that showed activity in preclinical models and in mice but has not yet been further investigated [36] and other monoclonal antibodies against FGFR4 are currently under development. A summary of currently open trials using FGF19 and FGFR4 inhibitors in various tumours can be found in Table 1.

**Table 1:** Open trials with FGF19 and FGFR4 inhibitors in various tumours.

Open Trials Targeting the FGF19/FGFR4 Pathway in Various Tumours	
AZD4547	AZD4547 & Anastrozole or Letrozole in ER+ Breast Cancer Patients Who Have Progressed on NSAIs (RADICAL)
AZD4547	Lung-MAP: S1400 Biomarker-Targeted Second-Line Therapy in Treating Patients with Recurrent Stage IIIB-IV Squamous Cell Lung Cancer
AZD4547	Intergroup Trial UNICANCER UC 0105-1305/ IFCT 1301: Efficacy of Targeted Drugs Guided by Genomic Profiles in Metastatic NSCLC Patients (SAFIR02_Lung)
AZD4547	Evaluation of the Efficacy of High Throughput Genome Analysis as a Therapeutic Decision Tool for Patients with Metastatic Breast Cancer (SAFIR02_Breast)
AZD4547	Open-Label, Randomised, Multi-Drug, Biomarker-Directed, Phase 1b Study in Pts w/Muscle Invasive Bladder Cancer (BISCAY)
JNJ-42756493	A Study to Evaluate the Safety, Pharmacokinetics, and Pharmacodynamics of JNJ-42756493 in Adult Participants with Advanced or Refractory Solid Tumors or Lymphoma
JNJ-42756493	An Efficacy and Safety Study of JNJ-42756493 in Participants with Urothelial Cancer
JNJ-42756493	Study to Evaluate the Safety, Pharmacokinetics, and Pharmacodynamics of JNJ-42756493 in Participants with Advanced Hepatocellular Carcinoma
FGF401	Safety and Efficacy of FGF401 in Patients with Solid Malignancies
BLU554	A Phase 1 Study of BLU-554 in Patients with Hepatocellular Carcinoma and Cholangiocarcinoma

## 5. Discussion

The need for more precise and personalized treatment for HCC is still unmet. Hepatocellular carcinogenesis is complex and different oncogenic pathways are involved. The lack of predictive biomarkers may well be the reason for the failure of various agents that have been used in the past.

Data for the role of FGF19/FGFR4 in liver oncogenesis is robust and oncogenomic screening has identified a subset of patients where this pathway appears to be the oncogenic driver. Several inhibitors of the pathway are under investigation and have shown promising results in the preclinical setting. Based on both *in vitro* and *in vivo* results it appears that a selective FGFR4 approach may be more promising for FGF19/FGFR4 driven HCC compared to pan-FGFR inhibitors. The optimal population for such a targeted approach needs yet to be defined. From existing data it seems that patients with tumours with detectable expression of FGF19, FGFR4 and Klothoβ would benefit from such an approach and approximately one third of all patients with HCC are expected to meet these criteria. It is critical that biomarker programmes are undertaken in parallel with clinical studies in order to identify predictive biomarkers that would enable us to select those patients that are more likely to benefit from such a therapeutic strategy.

FGFR4 inhibition is not expected to have the same toxicity profile with pan-FGFR inhibitors but may be associated with an increased risk of cholestatic injury in patients who already have dysregulation of bile acid homeostasis [57]. Many of the *in vivo* data so far are based on mice and the differences between mice and humans need to be taken into consideration before extrapolating these data to humans. For mice peak of cholesterol and bile acid synthesis is at midnight where in humans bile acid synthesis has two peaks per day and cholesterol synthesis follows a circadian rhythm [24].

## 6. Conclusions

Patients with advanced HCC yield a dismal prognosis and, unlike other cancer types, treatment options are extremely limited. The modest success of sorafenib has spurred on study of various targeted agents in this disease. However, all of the studies have been conducted in unselected populations without any predictive biomarkers which may be the reason for failing to show meaningful outcomes. It appears that a subset of tumours is driven by the FGF19/FGFR4 pathway and these patients could benefit from treatment with FGFR4 inhibitors. Several agents are under development and further research is eagerly awaited since it may provide a more effective treatment in one third of patients with HCC.
